# In Vitro Evaluation of the Antimicrobial and Immunomodulatory Activity of Culinary Herb Essential Oils as Potential Perioceutics

**DOI:** 10.3390/antibiotics9070428

**Published:** 2020-07-21

**Authors:** Marcela Popa, Luminița Măruțescu, Eliza Oprea, Coralia Bleotu, Crina Kamerzan, Mariana Carmen Chifiriuc, Grațiela Grădișteanu Pircalabioru

**Affiliations:** 1 Research Institute of University of Bucharest, University of Bucharest, 050095 Bucharest, Romania; bmarcelica@yahoo.com (M.P.); crina.saviuc@yahoo.com (C.K.); carmen.chifiriuc@gmail.com (M.C.C.); gratiela87@gmail.com (G.G.P.); 2 Organic Chemistry Department, Faculty of Chemistry, University of Bucharest, 050095 Bucharest, Romania; 3 Microbiology Department, Faculty of Biology, University of Bucharest, 050095 Bucharest, Romania; cbleotu@yahoo.com

**Keywords:** essential oils, antimicrobial activity, cytotoxicity, perioceutics, periodontal disease

## Abstract

Due to their antimicrobial, immunomodulatory, antioxidant, and regenerative activities, culinary herbs have multiple medicinal uses, among which to prevent and treat oral diseases. The whole essential oils (EOs) have multiple advantages over purified components, such as a low probability to select for antimicrobial resistance, synergic effects of different components, and multi-pharmacological activities. In this study, we aimed to evaluate essential oils from *Salvia officinalis* (sage), *Satureja hortensis* (summer savory), and *Anethum graveolens* (dill) using an in vitro analysis of their antimicrobial activity against Gram-positive and Gram-negative bacterial strains isolated from the oral cavity of patients with periodontitis; the assays addressed both the planktonic and biofilm growth states and used culture-based approaches. Some of the tested EOs exhibited excellent bactericidal and antibiofilm activity, being active at concentrations as low as 0.08–1.36 mg/mL. Flow cytometry was used to investigate the potential mechanisms of their antibacterial activity and confirmed that the tested EOs act by permeabilizing the bacterial membrane and by inhibiting the activity of the efflux pumps. The immunomodulatory effect of the three EOs was determined by analyzing the gene expression profiles for pro- and anti-inflammatory cytokines of the THP-1 cells. The summer savory EO induced a clear proinflammatory effect, while the others did not significantly influence the cytokines profile of the tested cells. Taken together, our results indicate that summer savory EO and, to a lesser extent, sage and dill EOs could be used to inhibit bacteria involved in oral plaque formation and to reduce the expression of genes known to contribute to the inflammatory response using cell culture assessment.

## 1. Introduction

Medicinal plants have been used for thousands of years around the globe, by around 80% of the world population, as traditional treatments for many ailments, particularly in developing countries [1]. Subsequent research has demonstrated that natural products derived from medicinal plants represent an abundant source of biologically active compounds and have been the basis for developing new chemicals for the pharmaceutical industry.

Currently, one of the most serious challenges of the pharmaceutical industry is the emergence of antimicrobial resistance, which is considered by World Health Organization (WHO) as one of the top ten global challenges [2]. Moreover, the adverse effects of currently used therapeutic agents (dysbiosis, hepato- and nephrotoxicity, and hypersensitivity reactions) has led to an increased interest in the discovery of new, natural compounds with anti-infectious and immunomodulatory activity.

Among these, essential oils (EOs), defined as volatile secondary metabolites of plants, have been extensively used in traditional medicine due to of their many different biological properties, including antimicrobial and immunomodulatory ones. EOs are produced by more than 17,500 species of plants from many angiosperm families, including *Asteraceae*, *Lamiaceae*, *Myrtaceae*, *Rutaceae*, and *Zingiberaceae*, but only a small fraction of them are commercially available [3]. EOs are a complex mixture of terpenes, terpenoids, and phenylpropanoids, but they may also contain sulfur derivatives, fatty acids, and oxides [4].

Periodontitis is the most common chronic infection in the adult population globally. It is estimated that about 50% of subjects affected have forms of gingivitis (inflammation of the bleeding gums with swelling, redness, exudate, and changes in the normal contour of insertion); 30% periodontitis (clinical semiology evolving towards progressive loss of the alveolar bone around the teeth); and 5–15% develop serious forms with ulceronecrotic gum damage and dental loss. Importantly, periodontitis represents a risk factor for serious systemic diseases, such as diabetes mellitus, obesity, and cardiovascular diseases [5,6,7], or even the development of tumoral processes [8,9,10]. It was reported that the subgingival plaque of patients with periodontitis is associated with a number of genera, including *Filifactor, Treponema, Porphyromonas, Tannerella, Eubacterium, Peptostreptococcaceae, Desulfobulbus, Lachnospiraceae, Mogibacterium, Alloprevotella, Hallella, Phocaeicola, Johnsonella*, and *Mycoplasma* [11]. Dental plaque is an oral biofilm that has a central role in the development of periodontitis. The most recent hypothesis explaining the involvement of dental plaque in periodontitis combines the concepts of the two earlier ones—respectively, the nonspecific and specific dental plaque hypotheses—and was called the “Ecological Plaque Hypothesis” (1994), proposing that the periodontal disease is the result of an oral microbiota dysbiosis, resulting in the selection of certain pathogenic microorganisms. This was changed into the “Keystone-Pathogen Hypothesis” (2012), proposing that certain low-abundant pathogens can cause inflammatory disease by interfering with the host immune system and remodeling the microbiota [12].

Existing treatments for the prevention and treatment of periodontal disease are expensive and have selective and time-limited efficiency. Mechanical or ultrasound-assisted debridement involving the use of surgical or nonsurgical techniques and the costs associated with surgical treatments have been estimated at $4–5000 per patient in the United States [13]. In this context, the identification of alternatives with preventive and/or therapeutic value for the management of periodontal disease is imperative.

“Perioceutics” involves the use of pharmacotherapeutic agents, including antimicrobial therapy and host modulatory therapy, specifically developed to manage periodontal disease [14]. Due to their complex composition of many active compounds with different targets, EOs seem to be a potential alternative to synthetic compounds, especially because of the resistance that has been increasingly developed by pathogenic microorganisms. Due to a high number of constituents, EOs can interact with multiple target sites, like the destruction of the cytoplasm membrane or inhibition of protein synthesis and efflux pump, etc. [15]. In the complex EO matrix, synergistic interactions between different components could occur. An example is offered by terpenes, which have a modest antimicrobial activity, but they favor the entry of terpenoids in the cell where they exhibit their antimicrobial effect. In addition, different terpenoids with aromatic structures could interact for the occurrence of the antimicrobial and antibiofilm activity [16]. Moreover, EOs are better tolerated in comparison with the synthetic antibacterial agents that are currently used for treatments of oral health problems, which are reported to cause several side effects such as diarrhea, vomiting, etc. [17]. EOs could also increase the susceptibility of microorganisms to antibiotics commonly used in dental practice and stimulate the beneficial host response. These are some of the advantages of using entire EOs instead of purified compounds. In this context, the aim of the present study was to investigate the bacterial growth inhibition, antibiofilm, and immunomodulatory effects of *Salvia officinalis* (sage), *Satureja hortensis* (summer savory), and *Anethum graveolens* (dill) essential oils against Gram-positive and Gram-negative bacteria isolated from the subgingival dental plaque of patients with periodontitis.

## 2. Results

### 2.1. Chemical Composition

Essential oils from *S. officinalis* (sage), *S. hortensis* (summer savory), and *A. graveolens* (dill) were obtained by hydrodistillation and further characterized by gas chromatography-mass spectrometry (GC-MS). In the case of *S. officinalis*, the GC-MS analysis led to the identification of 18 components, the major ones being α-thujone and camphor, with comparable percentages (25.778% and 26.316%, respectively). The results of the chromatographic analysis and the cumulative composition are given in Table S1.

In the case of *S. hortensis* L., the components identified by GC-MS analysis of the volatile oil extracted from leaves were carvacrol (54.069%), γ-terpinene (26.749%), and m-cymene (7.996%) (Table S1). The herbal EOs composition from four chemotypes of this species have been reported previously, containing specific combinations of components: (1) thymol and α-terpinene, (2) thymol and carvacrol, (3) carvacrol and α-terpinene, and (4) thymol [18]. Yazdanpanah and Mohamadi (2014) reported a similar composition for *S. hortensis* L. volatile oil extracted by hydrodistillation [19].

The composition of this volatile oil described in our study is within the limits of the four chemotypes described. However, the variability may also be explained by many other factors (the extraction method, the drying process of the plant material, etc.) [20]. The EO we extracted from *S. hortensis* leaves had a balanced composition with a terpenoid:terpene ratio of 54.66%:43.13%.

The compounds identified from the *A. graveolens* EO by GC-MS are listed in Table S1. All components of *A. graveolens* contain volatile oils, and their compositions are different depending on the vegetative/reproductive organ from which the extraction is carried out [21]. According to Rădulescu et al. (2010), the main components of the volatile oil extracted from the leaves of *A. graveolens* are α-felandren, limonene, and dill ether [22]. In our study, α-phellandrene (68.541%) and β-phellandrene (9.431%) were the main components identified in the *A. graveolens* EO.

The *A. graveolens* EO contained a higher amount of monoterpene hydrocarbons (87.834%) than *S. hortensis* and *S. officinalis* EOs, which had only 41.762% and 18.650%, respectively. In addition, phenylpropanoids (myristicin and apiol) were only identified in the *A. graveolens* EO, while the monoterpene ketones (camphor and α- and β-thujone) were highlighted only in EO extracted from sage, just as carvacrol (a phenol with a monoterpenic structure) was identified only the in *S. hortensis* EO.

### 2.2. Antimicrobial Activity

The antimicrobial activity of the volatile oils was evaluated against 25 Gram-positive and Gram-negative bacterial strains isolated from patients with oral diseases. These strains belong to species with proven pathogenic potential (*Streptococcus mitis, S. salivarius, Prevotella oralis, Fusobacterium mortiferum,* and *Actinomyces naeslundii*) or are members of the oral microbiota. The results are expressed as minimum inhibitory concentration—MIC values (mg/mL) established after the spectrophotometric reading of the optical density of bacterial cultures for the entire concentration range tested for each variant analyzed. The EOs were ranked according to their antimicrobial effect. The classification criterion was that proposed by Carson and Hammer [23]. Concentration ranges were established for the analytical variants: <1%, 1.5–2.5%, ~5%, and >5% (*v*/*v*). Thus, volatile oils with antibacterial activity at concentrations >5% were considered to have minimal effects (e.g., *A. graveolens*), and those with antimicrobial activity at concentrations <1% were considered to have maximum effects (e.g., *S. hortensis*).

The volatile oils from *S. hortensis* harbored the highest antimicrobial activity, with MIC values between 0.08 and 10.91 mg/mL. In the case of *Streptococcus* sp. strains, the *S. hortensis* extract exhibited MIC values between 0.17 and 2.72 mg/mL (Table 1).

The extract obtained from *A. graveolens* showed a variation in the bacterial growth inhibitory activity, with MIC values between 0.71–91 mg/mL. Half of the tested strains were susceptible at an MICs of a maximum 22.75 mg/mL. The composition of the *A. graveolens* EO is mainly monoterpenic, containing a high level of α- phellandrene (68.54%). The lower antimicrobial effect can be correlated with a low level of terpenoids in the *A. graveolens* oil.

Next, we have determined the minimal concentration for biofilm eradication (MBEC) for *S. officinalis, A. graveolens*, and *S. hortensis* EOs against a wide array of microbial strains isolated from patients harboring oral pathologies. Data are represented as mg/mL and are shown in Table 1.

We have observed that a higher concentration of the tested EOs is required for the destruction of the bacterial biofilm, in comparison with the planktonic cells. *S. hortensis* EO exhibited enhanced antibiofilm activity with MBEC concentrations under 1 mg/mL against *Pantoea* spp., *Fusobacterium mortiferum*, and *Actinomyces naeslundii* isolates.

### 2.3. Evaluation of the EO Effects on Bacterial Cells by Flow Cytometry

Flow cytometry (FC) in combination with two fluorochromes: propidium iodide (PI) and ethidium bromide (EB) were used for investigating the overall impact of the studied EOs on bacterial cells. The bacterial cultures belonging to the *S. salivarius* sp. *salivarius*, and *S. acidominimas* isolates grown in the presence of EOs at subinhibitory concentrations were analyzed by FC. The two strains were chosen due to their involvement in the first steps of dental plaque development and, also, because they are an important reservoir of antibiotic resistance genes in the dental biofilm. [24] The fluorescent nucleic acid stains PI and EB intercalates to DNA. When bound to DNA fluorescence, their fluorescence is enhanced ~10-fold.

PI is a membrane-impermeable dye that penetrates only cells with disrupted membranes, and as a consequence, the red PI fluorescence signal detected by the flow cytometer increases. Low-level fluorescence could be detected for the control viable cells. The red fluorescent signal of PI of the bacterial cell-exposed EOs, expressed as the median fluorescence intensity (MFI), was not significantly increased by comparison with the control, indicating the cells membranes were not compromised at EO subinhibitory concentrations.

The EB accumulation in bacterial cells exposed to subinhibitory concentration EOs was evaluated for the direct measurement of the inhibition of efflux at the single-cell level. The reduction in the median fluorescence intensity (MFI) of the bacterial cells correlated to the removal of the dye via efflux pumps [25]. Bacterial efflux pumps contribute to intrinsic resistance; typically, their overexpression confers a resistance to antibiotics, including fluoroquinolones, dyes (e.g., ethidium bromide), detergents (e.g., sodium dodecyl sulfate), and disinfectants. Genes encoding efflux pumps are usually chromosomally carried, ensuring the mutations among the community. Efflux inhibitors have been proven to be effective in suppressing bacterial virulence properties and antibiotic resistance [26,27].

**Figure 1:**
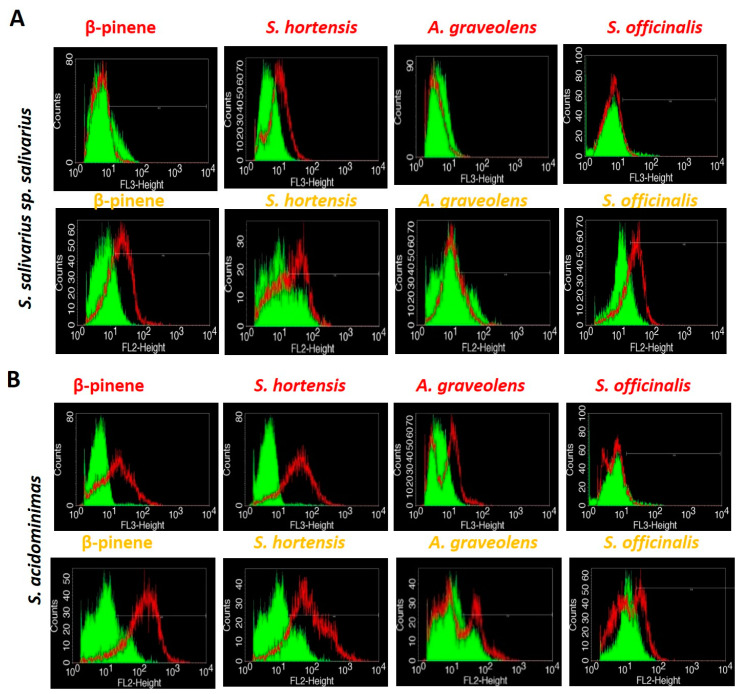
Histograms for *Streptococcus salivarius* sp. *salivarius* (panel **A**) and *S. acidominimas* (panel **B**) treated with volatile oils or analytical standard (β-pinene) vs. a viable cell control (marked with propidium iodide (PI) (red) and ethidium bromide (EB) (orange)).

Our study revealed that the tested EOs and the β-pinene analytical standard inhibited the activity of efflux pumps of *S. salivarius* sp. *salivarius* and *S. acidominimas* (Figure 1 and Table 2). The FC measurements showed an increase of more than two-fold of the MFI of the EO-treated bacterial cells, indicating that EB accumulated inside the bacterial cells. These results suggest that the EOs of *S. hortensis, A. graveolens*, and *S. officinalis* act as potential inhibitors of the *S. salivarius* sp. *salivarius* and *S. acidominimas* efflux pumps and could be considered promising candidates in developing potential adjuvant methods to therapeutically intervene against dental plaque biofilms. However, the FC analysis suggests that the mechanisms of action for these EOs are strain-specific, and further studies are required to address this issue.

### 2.4. Immunomodulatory Activity of the Tested EOs

The THP-1 cell line has become a model for the study of the functions and mechanisms of activation of monocytes and/or macrophages during the physiological or pathological inflammatory response, as well as of immunomodulators. THP-1 can be differentiated into macrophages using growth factors such as prostaglandin E2, phorbol 12-myristate 13-acetate, or lipopolysaccharides (LPS) [28]. LPS-treated THP-1 cells respond rapidly by altering the expression of interleukin genes (IL-1β, IL-6, IL-8, IL-10, and TNF-α), which can be detected rapidly after 1–6 h of incubation. In addition, the exposure of THP-1 cells to LPS results in the activation of transcription factor NF-κB, which, in turn, activates genes for other chemokines and cytokines involved in cell proliferation, differentiation, migration, and survival. In this study, we aimed to monitor the gene expression for pro- (IL-1β, IL-6, IL-8, and TNF-alpha) and anti-inflammatory (IL-10) cytokines in THP-1 cells treated with essential oils and then stimulated with LPS. Interleukin 10 is the main cytokine with anti-inflammatory effects, whose main function is to limit inflammatory reactions and regulate the differentiation and proliferation of immune cells (T, B cells, natural killer, antigen-presenting cells, mast cells, and granulocytes [29]. Out of the EOs tested, the *S. hortensis* volatile oil led to the upregulation of the IL-10 gene.

IL-1β, a proinflammatory cytokine, produced by both activated macrophages and a wide variety of other cell types, including B lymphocytes and endothelial cells, has pleiotropic effects and is involved in a variety of inflammatory reactions [30]. The tested extracts had various effects on interleukin-1β production. There is a strong stimulating (proinflammatory) effect of the essential oil extracted from summer savory, whereas the *A. graveolens* and *S. officinalis* EOs had no effect on *Il1b* expression.

**Figure 2:**
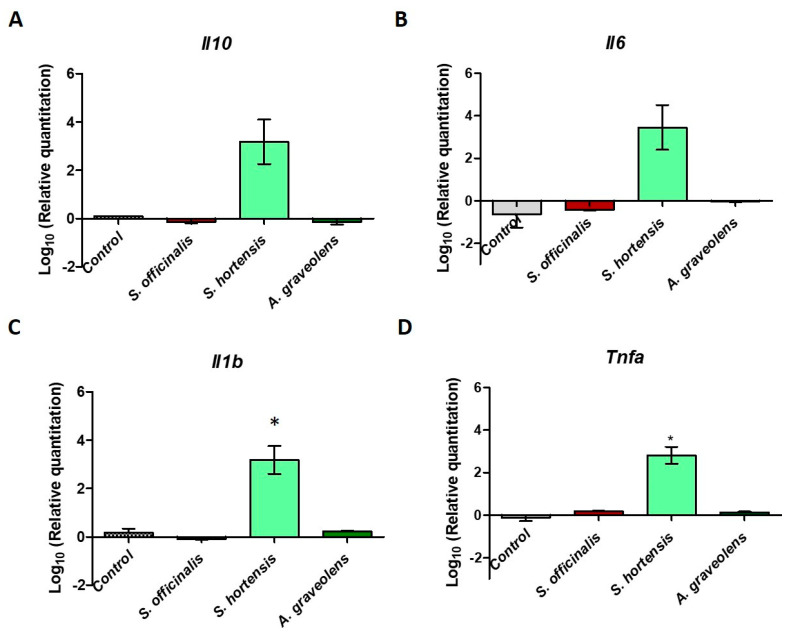
Cytokine expression in THP-1 cells treated with essential oils (EOs) extracted from *Salvia officinalis* (sage), *Satureja hortensis* (summer savory), and *Anethum graveolens* (dill). *n* = 3 (*t*-test) and control—unstimulated cells. (**A**): expression of IL-10; (**B**): expression of IL-6; (**C**): expression of IL-1β; (**D**): expression of TNF-α. *, significant difference *p* < 0.05.

IL-6 is a multifunctional cytokine secreted by monocytes, macrophages, T lymphocytes, endothelial cells, and fibroblasts in the early stages of the infectious process. IL-6 stimulates IgA synthesis in lymphoid structures associated with mucous membranes. The effects of IL-6 are synergistic with those of IL-1b, and it is an essential mediator of the acute phase response [31]. TNF-α is produced primarily by activated macrophages but, also, by other cells, including adipocytes, B lymphocytes, keratinocytes, fibroblasts, granulocytes, mast cells, monocytes, NK cells, smooth muscle cells, and T cells. IL-6 stimulates inflammation, under the action of variable stimuli, such as allogenic and bacterial cells, immune complexes, LPS, reactive oxygen species, malignant cells, and viral infections. *S. hortensis* volatile oil upregulated the gene expression for IL-6 and TNF-α, whereas the *S. officinalis* and *A. graveolens* volatile oils had no effect on cytokine gene expression (Figure 2B,D).

## 3. Discussion

The high incidence of oral diseases has triggered a global need for alternative prevention and treatment options that are both effective and economical. Despite some commercially available synthetic agents, these substances can alter the balance of the oral microbiota and have various local or systemic (e.g., vomiting, diarrhea, and staining of tooth enamel) side effects. For example, several antibacterial agents used in the prevention and treatment of oral conditions, including cetylpyridinium chloride, chlorhexidine, amino fluorides, or products containing such agents, are toxic and cause staining of the tooth enamel.

On the other side, there are approximately 500,000 plant species in the world, of which only 1% have been phytochemically analyzed, which means that there is a still a high potential for the discovery of new bioactive compounds [32]. Natural products derived from medicinal plants have proven to be an abundant source of pharmacologically active compounds, many of which being the basis for the development of synthetic or semisynthetic derivatives for the pharmaceutical industry. Volatile oils, products of plant secondary metabolisms, are valuable resources for the development of new anti-infective therapeutic alternatives. Numerous research papers but, also, patents are based on the use of volatile oils for various formulations with antimicrobial/antipathogenic activity.

Volatile oils have an antimicrobial activity positively correlated with the distribution of terpene:terpenoids in the complex mixture, in which synergism/antagonism interactions occur. Thus, the antimicrobial effect of volatile oils can be the result of a synergic activity of different components, influenced by specific environmental factors (pH, salinity, and temperature) or by the morphophysiological characteristics of the tested microbial strains (e.g., affinity for Gram staining). Many plant families are known to produce volatile oils: *Apiaceae, Asteraceae, Cupressaceae, Hypericaceae, Lamiaceae, Liliaceae, Myrtaceae, Pinaceae, Piperaceae, Rosaceae, Rutaceae, Fabaceae, Santalaceae, Zingiberaceae,* and *Zygophyllaceae.* They are used as a selection basis for alternatives to antibiotics/biocides currently used in medical practice. Carson and Hammer, 2011, mentioned that all volatile oils exhibit antimicrobial potential at concentrations not exceeding 5% (*v*/*v*) [33].

In the present study, most of the strains tested were susceptible to sage volatile oil at concentrations below 23% *v*/*v*. Its major components with known antimicrobial activity are the monoterpene ketones thujone and camphor, distributed in comparable percentages in the mixture (~ 25%). The volatile oil of sage was mostly made up of terpenoids (>80% of the total compounds identified). A study by Narayanan et al. (2015) revealed the antimicrobial activity of various extracts of *S. officinalis*, emphasizing that volatile oils harbor the highest activity [34]. Zeidán-Chuliá et al. reported the antibacterial properties of *S. hortensis L.* EO and *M. Salvia fruticosa* on *Fusobacterium nucleatum* and highlighted the fact that the strong inhibitory activity of *S. hortensis L.* is due to a higher concentration of terpenes [35].

The volatile oil obtained from aerial parts of summer savory—*S. hortensis*—was the most efficient in inhibiting the bacterial growth, with MIC values of 0.08 to 10.91 mg/mL. The major components in the volatile oil of *S. hortensis* were carvacrol and γ-terpinene, the distribution of terpenes:terpenoids being balanced in this mixture. Carvacrol is mentioned in the literature as a a substance with proven antimicrobial activity due to the presence of the free hydroxyl group, hydrophobicity, and the phenol moiety. Our results were similar to those of different research teams. Sharifi-Rad et al. used an extract from aerial parts of *Satureja intermedia C.A.* and reported MIC values of 4.2–5.1 mg/mL against *Streptococcus mutans* and an MIC of 11.4–12.5 mg/mL for *S. salivarius* [36]. Zomorodian et al. used extracts from *Satureja khuzestanica* and *Satureja bachtiarica* and reported MIC values between 0.062 and 0.25 and 0.125–0.5 µL/mL for a wide range of strains, including *S. mutans, S. sanguis, S. salivarius*, and *S. sobrinus* [37].

Zeidan-Chulia reported the antibacterial and antigelatinolytic activities of *S. hortensis* L. EO and recommended it for the potential treatment of periodontal inflammation. *A. graveolens* EO presented a variable inhibitory activity, most of the tested strains being susceptible at concentrations of 45.5 mg/mL [35]. The antimicrobial effect can be correlated with the poor representation of terpenoids in the mixture, the extract being mostly monoterpenic, with 68.54% α-felandren. Compared to *S. hortensis,* both *S. officinalis* and *A. graveolens* EOs were less efficient in inhibiting the bacterial growth.

A gradual accumulation of microbial plaque on the tooth surface represents one of the first stages of the dental caries process and periodontal diseases [38]. The microbial plaque is made up of a wide array of microorganisms characterized by complex interactions. In the early stages of dental plaque formation, Gram-positive cocci (*S. salivarius, S. sanguis*, *Streptococcus mitis,* and *S. salivarius)* attach to dental surfaces, followed by Gram-negative bacteria that complicate the plaque environment and increase tissue damage [39].

Dental conditions such as tooth decay and periodontitis are directly related to the ability of bacteria to form biofilms [40] in the form of supragingival and subgingival plaques. The development of dental caries implies the existence of Gram-positive acidogenic and aciduric species (*S. mutans, Lactobacillus* sp., and *Actinomycetes* sp.). On the other hand, periodontal lesions are associated with anaerobic Gram-negative bacteria (*Porphyromonas gingivalis, Actinobacillus* sp., *Prevotella* sp., and *Fusobacterium* sp.) [41]. The appearance and progression of these pathologies are influenced by the increased antibiotic resistance of bacterial biofilms [42]. With the increase in antibiotic resistance rates and the emerging resistance to conventional treatments, the discovery of novel agents targeting the microorganisms included in the biofilm is an important step in controlling oral cavity infections [43].

Despite the inhibitory activity of the EOs tested, the antibiofilm effect of *S. hortensis* EO was limited. Biofilm production of most of the strains was not inhibited by *S. officinalis* and *A. graveolens* EOs. This could be explained by the fact that bacterial cells incorporated into biofilms are more resistant to the action of conventional antibiotics compared to those in the planktonic state [42]. To explain the resistance of biofilms to antimicrobial substances, a number of mechanisms have been proposed: (1) restricted penetration of antimicrobial agents, (2) induction of the general stress response, (3) low growth and metabolic rates, (4) increased expression of multidrug efflux pumps, (5) activation of the quorum sensing and the response mechanism, and (6) modification of the profiles of external membrane proteins.

Different approaches have been employed to control the oral biofilm, such as preventing its formation and destroying the existing ones. However, the control of dental biofilms requires not only agents with bacteriostatic action but, also, antibiofilm activity to inhibit the initial adhesion of bacteria.

Plant-derived compounds and EOs can influence biofilm formations by inhibiting peptidoglycan synthesis [44], by damaging the bacterial membrane structure [45], and by modulating the quorum-sensing process [44].

In the present study, we evaluated the EO influence on membrane integrity and efflux pumps activity of the streptococcal strains, since it is well-known that *S. salivarius* is a reservoir of antibiotic resistance genes and exchanges genetic material with other species [46]. Integrative and conjugative elements are chromosomal mobile genetic elements detected in *S. salivarius* and in silico in other *Streptococcus* species (*S. pneumoniae* and *S. parasanguinis*) that can transfer autonomously by conjugation intra- and interspecies interactions [47]. In this approach, we have used a flow cytometry protocol, since is well-known that fluorescence-based flow cytometric methods could provide accurate, rapid, and highly sensitive results regarding the viability and vitality of the bacterial cells after incubation with EOs.

As the intensity of the inflammatory response induced by bacterial infections could be responsible of the severity of the tissue lesions, we have also tested how the EOs influenced the cytokine profiles of THP-1 cells. Among the tested oils, the *S. hortensis* oil exhibited an immunomodulatory effect characterized by an increased gene expression for both proinflammatory (IL-1, IL-6, and TNF) and anti-inflammatory (IL-10) cytokines, probably due to the increased cytotoxicity.

Thus, it can be concluded that phytochemical mixtures obtained from different plant species can be used in the treatment of pathological conditions to reduce or stimulate the occurrence of a proinflammatory responses, but their choice must be preceded by the correct assessment of the inflammatory status of the target tissue.

The results obtained regarding the antimicrobial and immunomodulatory potentials of these extracts recommend further research in order to obtain adjuvant or alternative strategies for the therapeutic and prophylactic management of periodontal diseases, as well as further studies to identify their molecular mechanisms of action.

## 4. Materials and Methods

### 4.1. Plant Material

The plant material was collected from Zorești, near Buzău, Romania, at the altitude of around 116 m (45°10′54″ N 26°42′10″ E) in late July 2014 by a local supplier. The aerial parts of *Salvia officinalis* and the leaves from *Satureja hortensis* and *Anethum graveolens* (separated from branches) were dried at room temperature and manually grounded. Their taxonomic affiliations were confirmed by Dr. Gheorghe Irina from the Botanical Garden “Dimitrie Brândză”, University of Bucharest.

### 4.2. Essential Oil Extraction

Isolation of essential oils was carried out using a standardized system of hydrodistillation in a Neo-Clevenger apparatus, described by various Pharmacopoeias (British Pharmacopoeia 2003) [48,49]. The essential oils were dried over anhydrous Na_2_SO_4_ and kept at 4 °C until analysis in dark glass bottles.

### 4.3. Chemical Characterization

For GC-MS analysis, the extracted volatile oil samples were diluted in hexane (5 µL/mL) and analyzed by gas chromatography coupled with mass spectrometry using the Agilent Series System 7890A-5975C analyzer. For the quantitative analysis, calibration curves were constructed for the analytical standards, and the concentration ranges used were established experimentally after the injection for the qualitative analysis of the volatile oils. The acquisition parameters used for the proposed acquisition method were: DB-5 MS chromatographic column (60 m × 0.25 mm × 0.25 µm-stationary phase made of poly-phenyl-arylene, nonpolar), temperature gradient elution, temperature program: 50 °C isotherm 3 min, 5 °C up to 300 °C, automatic injection in split mode, splitting rate 1:200, and injection volume 1 µL, flow rate 1 mL/min. The supplemental table lists the identified compounds in the essential oils from plants, retention times (expressed in minutes), and their relative areas (which are their areas reported to the total area of all compounds, expressed in percentages).

### 4.4. Antimicrobial Activity

Bacterial strains used in this study are members of the commensal oral microbiota and periodontal disease isolated from the subgingival bacterial plaque of individuals with periodontitis (from whom, written consent was obtained). In this study, we used 25 strains, identified by API Biomeriuex as *Enterococcus faecalis, Aerococcus viridans, Eubacterium lentum, Pantoea* sp., *Prevotela oralis, Actinomyces naeslundii, Gemalla morbillorum, Staphylococcus sciuri, Staphylococcus xylosus, Pasteurella haemolytica, Micrococcus* sp., *Fusobacterium mortiferum, Streptococcus salivarius* sp. *salivarius, Streptococcus intermedius, Streptococcus mitis,* and *Streptococcus acidomimimas.* The quantitative analysis of the antimicrobial activity of the volatile oils was performed using the method of serial microdilutions in a liquid medium (Mueller-Hinton broth) in 96-well plates [50,51]. The volatile oils and analytical standard (β-pinene was the chosen analytical standard, since it was found in all the EOs) were serially diluted in dimethylsulfoxide (DMSO) to a final volume of 200 µl. The negative control was represented by culture medium and the positive control by bacterial cultures. The plates containing the bacterial cells and the tested EOs were incubated at 37 °C for 18 h. The minimum inhibitory concentration (MIC) was established macroscopically as the last concentration at which the appearance of microbial growth was not observed, respectively, the appearance of turbidity and by spectrophotometric reading of the absorbance at 620 nm.

### 4.5. Antibiofilm Activity Testing

The EO influences on biofilm formations on inert substrata were assessed using the same procedure as the one for testing the antimicrobial activity, but after the incubation (18 h, 37 °C, no shaking), the plates containing the inoculum and the EOs were emptied of their contents, washed 3 times with saline solution to eliminate any nonadherent cells, and treated with alcohol in order to fix adherent cells. After violet crystal staining, the plates were treated with acetic acid 33% solution, and optical density was read at 490 nm using FlexStation3 (Molecular Devices, Foster City, CA, USA). Negative (culture medium) and positive (untreated cells) controls were used. The minimal biofilm eradication concentration (MBEC) corresponded to the last concentration at which there was no bacterial growth.

### 4.6. Evaluation of the EO Effects on Bacterial Cells by Flow Cytometry

The bacterial cells grown in the presence of the EOs were centrifuged at 12,000 rpm for 3 min, washed twice, resuspended in phosphate buffered saline (PBS) (adjusting, if necessary, the optical density OD_620_ of the suspended cells to 0.01–0.04 mau, corresponding to an inoculum under 10^7^ CFU/mL), stained (propidium iodide (PI 10 µg/mL) and ethidium bromide (EB 5 µg/mL), and incubated for 5 min at 4 °C in the dark. Positive (heat-inactivated cells at 100 °C for 1 h) and negative (viable cells) controls for membrane permeabilization were used. The fluorochromes used for cell staining allowed the investigation of the cellular coatings’ integrity (PI) and the efflux pumps’ activity (EB). For the assay, we used a FACS Calibur instrument equipped with a 488 nm Argon laser, using filters—a 670-nm-long pass-filter for samples stained with PI and a 585 ± 42-nm-band pass filter for the samples stained with EB. Forward scatter (FSC) vs. side scatter (SSC) and fluorescence measurements were acquired. Typical photomultiplier tube voltage parameters were SSC 550 V for bacteria and 450 V for fungal cells (log scale), a 670-nm-long pass filter (log scale) and a 585 ± 42-nm-band pass filter 550 V (log scale). A total of 10,000 events were collected in all runs.

### 4.7. Evaluation of the Immunomodulatory Activity

The THP-1 monocyte line (American Tissue Culture Collections–ATCC: TIB-202) (Rockville, MD, USA) was used for immunomodulation experiments. Cells were grown in RPMI 1640 medium with (Thermo Fisher Scientific, USA), 1.0 Mm sodium pyruvate, and 10% fetal bovine serum (FBS) (Thermo Fisher Scientific, USA). To evaluate the cytokine profile, THP1 cells (500,000 cells) were treated for 1 h with volatile oils (1:5000). After 1 h, LPS (1 µg/mL) was added, and incubation was continued at 37 °C for 3.5 h. Cells were harvested and centrifuged at 1300 rpm. Total RNA from cell sediments was extracted using an RNA isolation kit (RNeasy Mini Kit, Qiagen) according to the manufacturer’s instructions. The total RNA was eluted with 25 μL RNase-free water and DNase treated prior reverse-transcription (DNase TURBO, 2 Units, 30 min, 37 °C). The purity and concentration of RNA were established using a Nanodrop spectrophotometer, and the integrity of the total RNA was assessed through gel electrophoresis. Two micrograms of RNA from tissue samples was reverse-transcribed using the high-capacity cDNA reverse-transcription kit (Applied Biosystems, USA) according to the manufacturer’s protocol. The obtained cDNAs were subjected to real-time qRT-PCR (on a 7300 Applied Biosystems Real-Time PCR System) using the following TaqMan probes: Il-1beta (Assay ID: Hs00174097_m1); Il-2, Il-6, and Il-10 (Assay ID: Hs00174131_m1); TNF-a (Assay ID: Hs00174128_m1); and housekeeping gene TaqMan^®^ Ribosomal RNA Control Reagents (p/n4308313).

### 4.8. Statistical Analysis

Data regarding the immunomodulatory activity were analyzed by one-way ANOVA followed by a paired *t*-test using GraphPad Prism version 6.0 (GraphPad Software Inc., San Diego, CA, USA). Differences were considered significant at *p* < 0.05.

## 5. Conclusions

EOs harbor many different biological properties including antimicrobial and immunomodulatory activity. Plant-derived compounds can hamper biofilm formations by targeting peptidoglycan synthesis, by damaging the bacterial membrane or by inhibiting the quorum-sensing process. We show here that EOS from *Anethum graveolens*, *Salvia officinalis* and *Satureja hortensis* exhibited high bactericidal and antibiofilm activity against bacterial strains isolated from patients with oral diseases (*Streptococcus mitis, S. salivarius, Prevotella oralis, Fusobacterium mortiferum, Actinomyces naeslundii*). Subsequent flow cytometry analysis confirmed that the tested EOs act by permeabilizing the bacterial membrane and/or by inhibiting the activity of the efflux pumps. Out of the EOs tested, the summer savory EO induced a proinflammatory effect, while the others did not significantly influence the cytokines profile of THP-1 cells. Taken together, our results indicate that summer savory EO and, to a lesser extent, sage and dill EOs could be used to inhibit bacteria involved in oral plaque formation and to reduce the expression of genes known to contribute to the inflammatory response.

## Figures and Tables

**Table 1:** Antimicrobial activity of essential oil–minimum inhibitory concentration (EO–MIC) (mg/mL) values and minimal concentration for biofilm eradication (MBEC) (mg/mL) values.

Bacterial Strain	β-pinene		*Satureja hortensis* EO		*Anethum graveolens* EO		*Salvia officinalis* EO	
	MIC	MBEC	MIC	MBEC	MIC	MBEC	MIC	MBEC
S 39.1 *Enterococcus faecalis*	43.6	-	1.36	10.91	2.84	-	22.95	-
S 39.2 *Enterococcus faecalis*	5.45	5.45	5.45	21.82	91	-	22.95	22.95
S 26.4 *Enterococcus faecalis*	5.45	10.9	1.36	10.91	91	-	22.95	-
S 20.1 *Aerococcus viridans*	10.9	43.6	0.68	-	91	22.75	45.9	-
S 26.3 *Eubacterium lentum*	2.72	5.45	0.08	21.82	22.75	-	11.47	-
S 60.3 *Pantoea* spp.	21.8	21.8	0.17	0.84	11.37	22.75	5.75	45.9
S41.3 *Prevotella oralis*	0.68	0.68	0.68	10.91	1.42	2.84		-
S 41.1 *Actinomyces naeslundii*	5.45	5.45	1.36	10,91	45.5	-	22.95	22,5
S 41.2 *Actinomyces naeslundii*	21.8	10.9	0.34	21.8	22.75	22.75	22.95	2,86
S 114 *Actinomyces naeslundii*	5.45	10.9	2.72	0,68	22.75	22.75		-
S 61.2 *Gemella morbillorum*	5.45	21.8	1.36	21.8	22.75	22.75	11.47	45,9
S 60.1 *Staphylococcus sciuri*	5.45	10.9	0.34	1.36	5.68	-	11.47	5.73
S 119 *Staphylococcus xylosus*	5.45	21.8	2.72	2.72	45.5	-	45.9	45.9
S 42.1 *Gemella morbillorum*	1.36	21.8	0.34	-	45.5	2,84	45.9	-
S 50 *Pasteurella haemolytica*	10.9	10.9	0.68	5.45	45.5	11.37		22.95
S 66 *Pasteurella haemolytica*	21.8	21.8	1.36	21.8	91	22.75	22.95	45.9
S 65 *Micrococcus* sp.	10.9	10.9	10.91	10.91	22.75	-	45.9	-
S 71 *Fusobacterium mortiferum*	10.9	10.9	0.68	0,68	5.68	5,68	22.95	22.95
S 51 *Gemella morbillorum*	5.45	10.9	2.72	43.65	91	-	45.9	22.95
S 38.2 *Streptococcus salivarius* sp. *salivarius*	5.45	5.45	0.34	-	11.37	22.75	22.95	11.47
S 35.4 *Streptococcus intermedius*	21.8	21.8	1.36	1.36	0.71	-	45.9	-
S 47 *Streptococcus mitis*	5.45	10.9	0.34	5.45	45.5	-	45.9	
S 48 *Streptococcus acidominimas*	2.72	10.9	1.36	5.45	45.5	-	45.9	22.95
S 123 *Streptococcus acidominimas*	5.45	21.8	2.72	-	45.5	-	45.9	5.73
S 44 *Streptococcus acidominimas*	5.45	5.45	0.17	5.45	11.37	5.68	22.95	22.95

**Table 2:** Determination of the mechanisms of action of volatile oils and analytical standard β-pinene on the *Streptococcus* spp. by comparative analyses of the percentage increase in the median fluorescence intensity (MFI). PI: propidium iodide and EB: ethidium bromide, EP: efflux pumps, CWP: cell wall permeabilisation.

Strain	EO/Standard	MFI Sample (PI)	MFI Viable Cells Control (PI)	MFI Sample (EB)	MFI Viable Cell Control (EB)	MFI % (PI)	MFI % (EB)	Proposed Mechanism
*Streptococcus salivarius* sp. *salivarius*	β-pinene	5.47	4.52	18.43	6.92		166.33	EP
	*S. hortensis*	9.31	6.2	27.38	8.2	50.16	233.90	EP
	*A. graveolens*	3.79	6.2	10.05	8.2			
	*S. officinalis*	4	3.64	27.63	9.06		204.97	EP
*Streptococcus acidominimas*	*A. graveolens*	9.06	6.2	10	8.2	46.12		CWP
	*S. hortensis*	11.24	3.98	29.8	7.86	182.41	279.13	EP
	β-pinene	10.7	3.98	27.6	7.86	168.84	251.15	EP
	*S. officinalis*	5.8	3.64	12.63	9.06			

## Supplementary Materials

The following are available online at https://www.mdpi.com/2079-6382/9/7/428/s1. Table S1: Chemical composition of EOs by GC-MS.
