# Supplementary material for: In Vitro Evaluation of the Antimicrobial and Immunomodulatory Activity of Culinary Herb Essential Oils as Potential Perioceutics
Source: Antibiotics (Basel). 2020 Jul 21;9(7):428. doi: 10.3390/antibiotics9070428 (PMC7399839; doi:10.3390/antibiotics9070428)
Supplement: Supplementary file 1 [file antibiotics-09-00428-s001.pdf]

Table S1. Chemical composition of EOs by GC-MS.

No.	Compound	Retention time (min)	Relative area (%)		
			Salvia officinalis EO	*Satureja hortensis* EOEO	*Anethum graveolens* EO
**1**	α-pinene	7.39	6.850	1.104	2.007
**2**	camphene	7.675	7.051	0.114	-
**3**	β-myrcene	7.977	0.592	1.566	-
**4**	β-pinene	8.064	0.818	0.455	0.468
**5**	α-phellandrene	8.397	-	0.348	68.541
**6**	α-terpinene	8.541	-	2.927	-
**7**	m-cymene	8.656	-	7.996	-
**8**	o-cymene	8.746	1.501	-	4.289
**9**	d-limonene	8.832	1.838	0.503	3.098
**10**	β-phellandrene	8.903	-	-	9.431
**11**	γ-terpinene	9.142	-	26.749	-
12	cis-terpineol	9.371	-	0.166	-
13	1,8- cineole	9.941	8.262	-	-
14	α-thujone	10.061	25.778	-	-
15	β- thujone	10.224	7.778	-	-
**16**	camphor	10.798	26.316	-	-
**17**	iso-thujol	10,969	0.392	-	-
18	terpinen-4-ol	11.138	0.410	0.249	-
**19**	borneol	11.144	2.157	-	-
**20**	bornyl acetate	12.757	0.947	0.176	-
**21**	carvacrol	12.857	-	54.069	8.625
**22**	β-caryophyllene	15.378	-	0.427	-
**23**	alfa-caryophyllene	16.028	0.387	-	-
**24**	muurolene	16.105	-	0.138	-
**25**	β-bisabolene	16.442	-	0.59	-
**26**	α-cadinene	16.687	-	0.214	-
27	myristicin	16.711	-	-	0.468
28	epiglobulol	17.944	2.827	-	-
29	apiol	17.977	-	-	1.106
30	epimanool	22.327	0.580	-	-
31	adipic acid ester 1	22.179	5.525	-	-
32	adipic acid ester 2	25.176	-	-	0.46
